# Impact of Camera Settings on 3D Reconstruction Quality: Insights from NeRF and Gaussian Splatting

**DOI:** 10.3390/s24237594

**Published:** 2024-11-28

**Authors:** Dimitar Rangelov, Sierd Waanders, Kars Waanders, Maurice van Keulen, Radoslav Miltchev

**Affiliations:** 1 Technologies for Criminal Investigations, Saxion University of Applied Sciences, 7513 AB Enschede, The Netherlands; d.g.rangelov@utwente.nl (D.R.); s.c.waanders@saxion.nl (S.W.); k.t.waanders.01@saxion.nl (K.W.); 2 Faculty of Electrical Engineering, Mathematics and Computer Science, University of Twente, 7522 NB Enschede, The Netherlands; 3 Police Academy of the Netherlands, 7334 AC Apeldoorn, The Netherlands; 4 Faculty of Industrial Technology, Technical University of Sofia, 1756 Sofia, Bulgaria; rmiltchev@tu-sofia.bg

**Keywords:** crime scene reconstruction, 3D reconstruction, Neural Radiance Fields, crime investigation, 3D scanner technology, forensic photogrammetry

## Abstract

This paper explores the influence of various camera settings on the quality of 3D reconstructions, particularly in indoor crime scene investigations. Utilizing Neural Radiance Fields (NeRF) and Gaussian Splatting for 3D reconstruction, we analyzed the impact of ISO, shutter speed, and aperture settings on the quality of the resulting 3D reconstructions. By conducting controlled experiments in a meeting room setup, we identified optimal settings that minimize noise and artifacts while maximizing detail and brightness. Our findings indicate that an ISO of 200, a shutter speed of 1/60 s, and an aperture of f/3.5 provide the best balance for high-quality 3D reconstructions. These settings are especially useful for forensic applications, architectural visualization, and cultural heritage preservation, offering practical guidelines for professionals in these fields. The study also highlights the potential for future research to expand on these findings by exploring other camera parameters and real-time adjustment techniques.

## 1. Introduction

Three-dimensional (3D) reconstruction is the process of creating a three-dimensional model of a real-world object or scene from a series of two-dimensional (2D) images. This provides detailed and precise digital representations for various applications. It is crucial in fields such as engineering, medicine, robotics, city planning, archaeology, and many more, where high-fidelity spatial data are essential for analysis and decision-making [1].

One field that can greatly benefit from detailed 3D reconstruction is crime investigation [2,3,4,5,6]. It is a topic widely discussed in the field of forensics. The primary aim in this context is to reconstruct and understand what happened at a crime scene. Investigators collect as much evidence as they can to support or challenge their hypotheses, aiming to answer key questions of the investigation. This task is challenging because only the results and aftermath of a crime are evident, leading to various interpretations. Investigators must identify and analyze both obvious and hidden evidence to determine its origins and reasons. However, traces alone are insufficient to fully outline the sequence of events. They provide mainly hints and directions. Making sense of these hints requires interpretation, connecting them to actions, and creating a narrative about the crime. The integration of 3D reconstruction techniques into forensic investigations has significantly enhanced the analysis and interpretation of crime scenes. By creating detailed digital models, investigators can preserve spatial relationships and visual details that might be missed with traditional methods. These reconstructions provide a comprehensive, interactive view of the scene, aiding in connecting evidence to actions and facilitating a more accurate understanding of events. For instance, a study demonstrated the application of virtual 3D multimodal approaches to victim and crime scene reconstruction, highlighting the potential of these technologies in forensic science [7].

Our previous research has already explored the impacts, benefits, and limitations of 3D reconstruction technology in crime scene investigations, demonstrating its transformative potential to enhance efficiency, accuracy, and safety in forensic workflows. However, to fully harness its capabilities, challenges such as ethical considerations and technical optimization must be addressed [8].

In this research, we focused on the application of 3D reconstruction to indoor crime scene investigations, specifically examining how various camera settings impact the quality of the reconstructions. Our findings, while tailored to crime scene investigations, are also directly applicable to other fields requiring high-quality 3D modeling, such as architecture, cultural heritage preservation, and virtual reality (VR) environments. By identifying optimal camera settings that enhance detail, reduce noise, and accurately capture lighting, we offer practical insights that can improve 3D modeling processes in these areas. This paper demonstrates how these optimized settings can lead to better results, not only in forensic contexts but also in a range of other professional applications where precision and realism are critical.

For example, architectural visualization can benefit from accurate indoor 3D models for design and renovation projects [9,10,11]. In cultural heritage preservation, detailed reconstructions of historical sites and artifacts can aid in documentation and restoration efforts [12,13]. Similarly virtual reality (VR) and augmented reality (AR) applications in gaming and simulation training can achieve higher realism and immersion with optimized 3D reconstruction techniques [14].

To achieve these improvements in 3D reconstruction, we utilized Neural Radiance Fields (NeRF) [15], a relatively new approach that uses deep learning to generate highly detailed and accurate 3D models from 2D images. This method leverages the spatial and light interactions within the captured images to produce realistic 3D representations. Gaussian Splatting [16] further enhances this process by refining and smoothing the spatial data points, which leads, therefore, to better quality of the reconstruction.

There are multiple ways to conduct 3D reconstruction. Each has unique strengths and applications. The traditional methods are photogrammetry and structured light scanning.

- *Photogrammetry* is a methodology that involves taking multiple pictures of an object from different angles and using software to reconstruct a 3D model from these images. This method is widely used because it is both simple and effective. It is especially valuable in fields like archaeology and cultural heritage preservation, where non-invasive techniques are essential to avoid damaging artifacts [17,18,19,20].
- *Structured light* scanning projects a series of light patterns onto an object and captures the deformation of these patterns with a camera. The distortions in the light patterns are then used to calculate the object’s 3D shape. This method provides high accuracy and is commonly used in industrial applications for quality control and reverse engineering, where precision is paramount [21,22,23].

NeRF, however, represents a significant breakthrough in this area. NeRF uses deep learning to generate highly detailed 3D models from 2D images. It leverages the spatial and light interactions within the captured images to produce realistic and accurate 3D representations. It has shown quite remarkable results in the resolution of fine details and handling complex lighting conditions. Thus, it is applicable for use in operations that require high precision. NeRF is not the first step in this new evolution of 3D reconstructions but is, rather, the building block of a family of algorithms, which includes SNeRF [24], Tetra-NeRF [25], NeRFacto [26], Instant-NGP [27], SPIDR [28], MERF [29], and so on. In fact, each one of them solves particular problems and allows for the increment of overall capabilities in different ways.

LumaAI [30] provides a framework for building high-quality 3D reconstructions using the NeRF technology. For this research, we utilized LumaAI due to its robust features and ease of use.

The quality of 3D reconstructions strongly depends on the camera settings during image capture. The most important settings include ISO, shutter speed, and aperture. These factors determine sharpness, brightness, and detail in taking photographs, which determine the quality of a 3D model.

- ISO represents the sensitivity of the camera sensor to light. Higher ISO values increase the camera’s sensitivity, allowing for better performance in low-light conditions, but they can also result in a noisier image.
- Shutter speed defines the length of time that the camera’s sensor is exposed to light. Fast shutter speeds freeze motion and reduce blur, while slow shutter speeds allow more light to enter but may cause motion blur.
- The aperture controls both the brightness of the light reaching the sensor and the depth of field in the image. A larger aperture (indicated by a smaller f-number) results in a shallower depth of field and lets in more light, whereas a smaller aperture (indicated by a larger f-number) provides a greater depth of field but lets in less light.

The type of camera also plays a significant role. Full-frame cameras have a larger sensor, thereby capturing much more light resulting in higher quality image reception, particularly in low-lighting conditions. They are broader in the field of view and afford a better degree of control in respect to depth of field over cropped sensor cameras. However, cropped sensor cameras, or APS-C cameras, are much cheaper and quite small in size, which makes them very practical in most applications [31,32]. Depending on the specific needs and constraints of the 3D reconstruction task at hand, each type of camera has its own strengths.

Accurate 3D reconstructions in crime scene investigations may provide crucial insights into the sequence of events and assist investigators in analyzing evidence more efficiently. Traditional methods, such as sketches or photographs, often lack the spatial depth and context needed for a complete understanding of the crime scene, which can lead to false interpretations. For example, in a case involving a gunshot, a 2D photograph might show the bullet hole in the wall but may fail to provide enough spatial information about the angle and distance from the shooter’s position, potentially leading to an incorrect interpretation of the bullet’s trajectory or direction. In contrast, a 3D reconstruction captures the precise spatial relationships between the bullet hole, the possible shooter’s position, and other elements in the scene, allowing for a more accurate analysis of the evidence. This technology not only helps avoid misinterpretations but also provides an interactive tool for investigators and legal professionals to visualize and communicate the crime scene, aiding in courtroom presentations and ensuring that key evidence is clearly understood.

This paper contributes primarily to the field of 3D reconstruction with a special emphasis on its application within crime scene investigation. The contributions can be divided into theoretical and practical advancements.

**Theoretical Contributions**

- **Impact analysis of camera settings:** This study presents an in-depth theoretical analysis of how various camera settings, ISO, shutter speed, and aperture, affect the quality of 3D reconstructions. It enhances the understanding of how these settings influence noise levels, detail accuracy, and brightness in the resulting models, providing a clear link between camera configuration and model fidelity.
- **Advancement in 3D reconstruction techniques:** We specifically advance two techniques, NeRF (Neural Radiance Fields) and **Gaussian Splatting**, demonstrating their effectiveness in generating highly detailed and accurate 3D models from 2D images. These techniques excel in addressing challenges posed by complex lighting conditions and fine details, making them particularly well-suited for scenarios requiring high precision, such as crime scene investigations.
- **Extension of theoretical framework:** This research expands the theoretical framework for optimizing camera settings in 3D reconstruction across diverse fields, such as architecture, archaeology, and digital media. By applying advanced techniques like NeRF and Gaussian Splatting, the study shows the potential for significant improvements in accuracy and detail. The findings lay the groundwork for future research in extending 3D reconstruction methodologies to new applications and environments.

**Practical and technical contributions**

- **Crime Scene Investigation Guidelines:** The findings of this research provide guidelines for forensic investigators to obtain accurate, detailed 3D reconstructions of crime scene investigations. The survey further points out ISO 200 and 1/60 s as the speed at which the shutter should operate, while the aperture should be f/3.5, at a good average rate that balances noise, detail, and brightness.
- **3D Modeling Processes Optimization:** The results of this research offer practical recommendations for optimization of 3D modeling processes within numerous fields. These recommendations can significantly improve the quality of 3D models used in architectural visualization, cultural heritage preservation, and VR/AR applications for enhanced realism and immersion.
- **Multi-technique Camera Movement Strategy for Enhanced Data Collection:** A multi-technique camera movement strategy is introduced, combining truck, pedestal, boom, and arc techniques to ensure comprehensive scene coverage and capture essential spatial and lighting details. This strategy improves the accuracy and depth of 3D reconstructions, benefiting fields such as architecture, cultural heritage preservation, and VR/AR applications.

This paper advances the methodology of 3D reconstructions in crime scene investigation with both theoretical and practical contributions, thereby widening the scope of applicability of 3D reconstruction technology in many professional fields. Here, we deal with the influence of selected camera settings on 3D reconstruction quality for indoor criminal situations. By optimizing such settings, the quality of 3D models and the suitability for use as tools of trade, can potentially be improved.

This paper is organized as follows: Section 2 describes the methodology used in this study, including the technical parameters employed during image capture and how they meet the demands of crime scene investigations. Section 3 presents the results of our experiments and comparative analysis of different camera settings. Section 4 provides a detailed discussion of these findings, comparing them with the existing literature and exploring their implications for various applications. Finally, Section 5 concludes the paper, summarizing our key contributions and suggesting directions for future research.

## 2. Materials and Methods

### 2.1. Experimental Setup

The experimental setup consists of a meeting room with several key features, such as a table, an image of a man on the wall, whiteboard, ceiling, and lamp. These elements were chosen to provide a diverse set of objects with different geometric and textural information for NeRF.

The primary equipment used was a Canon 70D DSLR [33] camera equipped with a 22.5 × 15 mm CMOS sensor (Canon Inc., based in Tokyo, Japan). This camera was chosen for its balance of high image quality and manual control options, which are crucial for precise adjustments of ISO, shutter speed, aperture, and resolution. The lens combined with the camera is a Canon EF-S 18–55 mm lens. A controlled lighting condition was maintained across all captures. The EOS 70D offers an ISO range from 100 to 12,800, expandable to 25,600, and a shutter speed range from 1/8000 to 30 s, including a Bulb mode for extended exposures. Aperture settings depend on the attached lens. With the Canon EF-S 18–55 mm f/3.5–5.6 IS STM lens (Canon Inc., based in Tokyo, Japan), the aperture ranges from f/3.5 at 18 mm to f/5.6 at 55 mm. The camera captures images at a maximum resolution of 5472 × 3648 pixels. No hardware or software image stabilization systems were employed during data collection.

To improve the quality of 3D reconstructions, particularly in noise reduction, detail accuracy, and brightness control, we used Luma AI, a web-based implementation of Neural Radiance Fields (NeRF). The goal was to optimize key camera settings, ISO, shutter speed, and aperture, while generating accurate 3D models of a stagged indoor crime scene.

We conducted three comparison experiments, each varying one parameter at a time, using a consistent path and camera movement through the room. The video footage was processed into 3D reconstructions, which were evaluated based on noise, detail accuracy, and brightness relative to the actual environment. These criteria were chosen for their importance in forensic applications where both clarity and realism are essential for analyzing evidence.

This approach allowed us to identify the optimal camera settings for high-quality 3D reconstructions in indoor crime scene scenarios, providing valuable insights for future forensic investigations.

### 2.2. Camera Settings

The quality of a 3D reconstruction is significantly influenced by various camera settings. For this study, we focused on three primary parameters: ISO, shutter speed, and aperture. These parameters manage light interacting with the camera sensor and the detail in the images, thus impacting clarity, brightness, and overall quality of the 3D model.

- **ISO:** ISO measures the camera’s sensitivity to light. Higher ISO values allow for better performance in low-light conditions but can introduce noise into the images. Lower ISO settings produce cleaner images but require more light.
- **Shutter Speed:** Shutter speed refers to how long the camera’s shutter remains open to allow light to hit the sensor. Faster shutter speeds can freeze motion, reducing blur, while slower speeds allow more light but can cause motion blur.
- **Aperture:** The aperture is the opening in a lens through which light passes to enter the camera. A wider aperture (lower f-number) results in a brighter image and a shallower depth of field, while a narrower aperture (higher f-number) provides a greater depth of field but reduces light intake.

The parameters were varied across different tests to evaluate their impact on 3D reconstruction quality. The specific settings used for each comparison are detailed in the tables below (Table 1, Table 2 and Table 3).

By systematically varying these parameters and analyzing the resulting 3D reconstructions, we aimed to identify the optimal settings for high-quality 3D modeling in indoor crime scene scenarios.

### 2.3. Data Collection

The camera was handheld throughout the capture process, allowing for flexible, controlled movements as needed. We employed four distinct camera movement techniques, truck, pedestal, boom, and arc, to ensure comprehensive coverage of the scene and high-quality data for the 3D reconstruction. Each technique was strategically used at different points along the path based on the spatial characteristics of the scene and the specific details we aimed to capture.

- **Truck**—This technique involves moving the camera horizontally left or right while maintaining a constant level. The lateral movement encapsulates the scene through different horizontal angles, thus covering a wide field of view.
- **Pedestal**—The camera is moved upwards or downwards without changing its angle. This is very similar to the boom, with the only difference being that the camera remains level. This is very useful to maintain a roughly similar viewpoint within the scene and at the same time capture any vertical variations that may exist on end.
- **Boom**—A crane shot that moves the camera vertically up or down, often combined with a tilting motion. This combined action allows the camera to adjust its angle while changing height, capturing varying perspectives and angles within the frame. This technique adds depth.
- **Arc**—The camera is moved in a circular motion on the horizontal axis. This movement is useful when encountering a corner in a space or when walking around an object.

**Figure 1:**
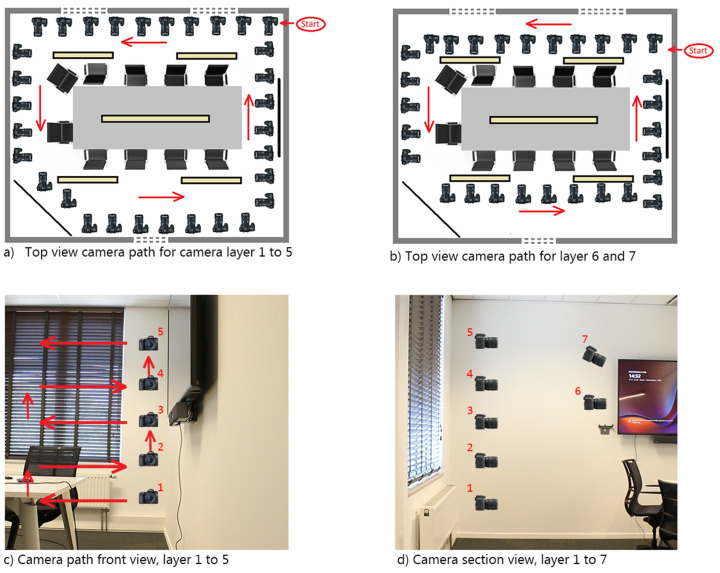
Data collection method (**a**) Top view camera path for camera layer 1 to 5, (**b**) Top view camera path for layer 6 and 7, (**c**) Camera path front view, layer 1 to 5, and (**d**) Camera section view, layer 1 to 7.

These techniques were not used for comparison but combined into one capturing method to complement each other during the capturing process. Each technique was applied based on the specific spatial requirements of the scene. The capturing process is visualized in Figure 1a,b, which illustrates the walking path through the scene, showing how the different techniques were applied at various points along the path. Figure 1b shows the close-up capturing path of the meeting table. This multi-technique approach, combined with the strategic use of camera movement, ensures a comprehensive loop closure and helps achieve an accurate and high-quality 3D reconstruction. Figure 1c,d display the different positions of the camera.

In the setup, only electric lamps were used to maintain a controlled lighting environment, ensuring consistency across captures. While the findings in this study are specifically based on controlled electric lighting, they have potential applicability in daylight or LED lighting conditions. However, further research is necessary to evaluate and compare results across different lighting types. For this paper, the experiments were conducted exclusively under controlled electric lamp lighting to ensure precision and reproducibility.

Each capture session lasted approximately 5–6 min, allowing for quick repetitions if necessary to ensure high-quality results. This approach balances the need for superior image quality with the practical demands of forensic applications.

### 2.4. Comparative Analysis Criteria

In this study, we captured a continuous video sequence using a handheld camera, which was then processed into a 3D reconstruction model. Although the data were captured as video, we evaluated specific frames to assess critical aspects of the 3D model. These frames were chosen for their ability to represent different textures, lighting conditions, and surfaces, common in indoor crime scenes.

We focused on two areas: a table with a TV and a whiteboard. These areas offer varying textures and lighting challenges, making them ideal for comparison. The 3D reconstructions were evaluated based on three criteria: (1) Noise (artifacts in the model), (2) Details (fidelity of the reconstruction), and (3) Brightness (accuracy compared to actual lighting).

The Canon EOS 70D captures images at a maximum resolution of 5472 × 3648 pixels, providing high-quality imagery suitable for detailed analysis. While our primary focus was on brightness, noise, and detail fidelity, we recognized that resolution is also crucial for forensic reconstruction applications. The high-resolution capabilities of the Canon EOS 70D ensured that the captured images met the stringent requirements necessary for such detailed forensic analyses.

Though literature on 3D reconstruction evaluation exists, most of it focuses on spatial accuracy and resolution. Our study introduces brightness as a key evaluation factor, particularly important for realistic crime scene reconstructions. In Table 4, these criteria are rated on a 1–5 scale, allowing us to systematically compare different camera settings. This framework adds a novel approach for assessing 3D reconstructions in forensic applications.

## 3. Results

This section presents the findings from the comparative analysis of different camera settings on 3D reconstruction quality. The aim is to obtain insights about optimal settings for high-quality 3D models, particularly indoors and for crime scene investigation.

### 3.1. Comparison 1: Impact of ISO Settings on 3D Reconstruction Quality

The first comparison focused on evaluating the impact of different ISO settings on 3D reconstruction quality. ISO measures the sensitivity of the camera’s sensor to light, with higher ISO values allowing better performance in low-light conditions but introducing more noise into the images. Conversely, lower ISO values produce cleaner images but require more light. The comparison results are shown in Table 5.

When evaluating the amount of noise and artifacts, the reconstructions varied significantly. At ISO 100, the image was notably pixelated, scoring a 1, indicating a high level of noise. The ISO 200 setting improved the situation with a score of 2, showing moderate noise levels. Similarly, ISO 400 maintained the score of 2, suggesting a comparable level of noise to ISO 200. However, ISO 800 also scored a 2, indicating that while the noise increased from ISO 200 to ISO 400, it did not worsen further at ISO 800 despite higher sensitivity. This suggests that ISO settings of 200 or higher are optimal in terms of managing noise while maintaining acceptable image clarity.

In terms of detail, there were clear differences across the ISO settings. ISO 100 scored a 2, showing pixelation but still allowing for some discernment of object types. ISO 200 improved to a score of 3, where object identification was easily achievable. At ISO 400, the detail was compromised by noise, resulting in a score of 2. Similarly, at ISO 800, the high noise levels also resulted in a score of 2. Thus, ISO 200 provided the highest level of detail, making it the most effective setting for capturing finer features.

For brightness, the variations were particularly notable. ISO 100 resulted in an underexposed image, scoring a 1. The ISO 200 setting provided a significantly better representation, closely matching the actual room brightness and scoring a 4. ISO 400 achieved the best brightness, scoring a 5, suggesting that it most accurately reflected the room’s lighting conditions. ISO 800, however, resulted in an overly bright image, bringing the score down to 2. Hence, ISO 400 was optimal for brightness, with ISO 200 also performing well.

Taking all criteria into account, reconstructions at ISO 200 demonstrated the best overall quality. While both ISO 200 and ISO 400 maintained a moderate level of noise (score of 2), ISO 200 excelled in detail (score of 3) and brightness (score of 4). ISO 400, although performing well in brightness (score of 5), was less effective in capturing details (score of 2). Conversely, ISO 100 suffered from high noise and poor brightness, while ISO 800 was marred by excessive noise and overexposure.

**Figure 2:**
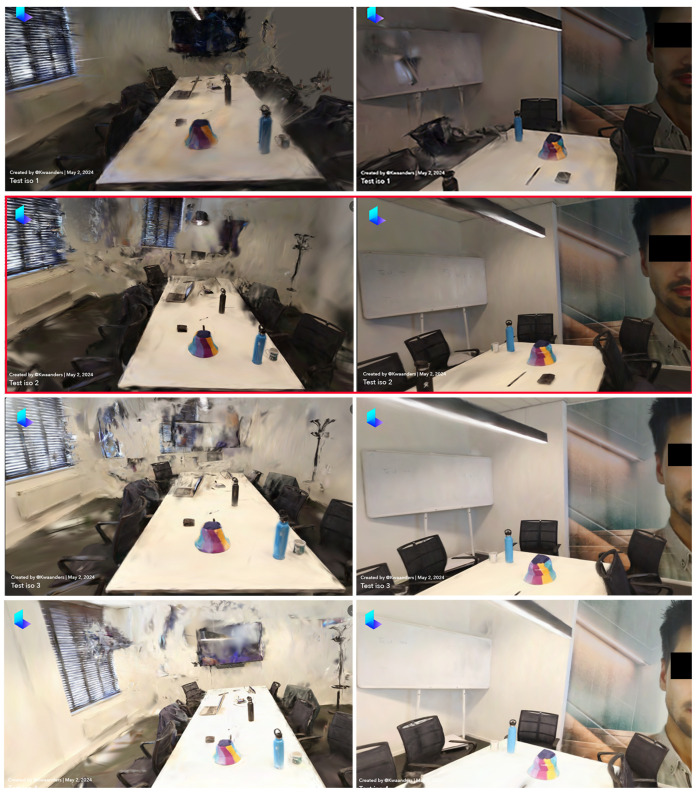
ISO comparison—(**1**) ISO 100, (**2**) ISO 200, (**3**) ISO 400, (**4**) ISO 800.

In summary, ISO 200 (Table 5) provided the best balance between noise, detail, and brightness, making it suitable for high-quality 3D reconstruction of the indoor environment. ISO 400, while offering excellent brightness, fell short in detail due to noise. Therefore, for optimal results in crime scene investigations and other detailed 3D modeling applications, an ISO setting of 200 is recommended., providing practical guidelines for crime scene investigations and other applications requiring detailed 3D modeling, as presented in Figure 2.

### 3.2. Comparison 2: Impact of Shutter Speed Variations on 3D Reconstruction Quality

This section delves into the impact of different shutter speeds on the quality of 3D reconstructions. Shutter speed refers to the amount of time that the camera’s sensor is exposed to light. Faster shutter speeds can freeze motion, reducing blur but allowing less light, while slower shutter speeds increase light intake but can introduce motion blur. The shutter speeds tested in this comparison were 1/30, 1/60, 1/125, and 1/250 s, with all other parameters kept constant (ISO 400, aperture f/4, and 24 FPS). The reconstructions were assessed based on three criteria: the noise in the image, the level of detail in the reconstruction, and the brightness of the reconstruction compared to the actual room brightness, as shown in Table 6.

When analyzing the amount of noise in the images, significant differences emerged among the reconstructions. The reconstructions at 1/30 and 1/250 s had a lot of noise, scoring 2 and 1, respectively. These images contained excessive noise, making it difficult to visualize the entire room accurately. Conversely, the reconstructions at 1/60 and 1/125 s were nearly noise-free, scoring 4 and 5, respectively. These images showed minimal artifacts, with the walls and other surfaces being clear and unobstructed. Therefore, in terms of noise, the reconstruction at 1/125 s was superior, with 1/60 s also performing well.

The level of detail varied distinctly across different shutter speeds. The reconstructions at 1/30 and 1/250 s lacked detail, with most items obscured by noise, resulting in scores of 3 and 2, respectively. While the text on the whiteboard was visible in both cases, finer details such as the text on the coffee mug and parts of the TV, were lost. In contrast, the reconstructions at 1/60 and 1/125 s were of much better quality, scoring 5 in both cases. These images allowed for clear identification of objects and even small text, indicating that these shutter speeds are optimal for capturing detailed 3D models.

Brightness was another crucial factor in the analysis. The reconstruction at 1/250 s was excessively dark, scoring a 1. The reconstruction at 1/125 s was also on the darker side, scoring a 3. In contrast, the reconstructions at 1/30 and 1/60 s closely matched the actual room brightness, both scoring 4. While 1/30 s was slightly lighter and 1/60 s slightly darker, both were close to the actual environment’s brightness levels. Therefore, in terms of brightness, both 1/30 and 1/60 s were effective.

Considering all three criteria, reconstructions at 1/60 and 1/125 s emerged as the highest quality. While both had minimal noise and high detail levels, 1/60 s provided better brightness, making it slightly superior overall. Conversely, reconstructions at 1/30 and 1/250 s were marred by excessive noise and poor brightness, making them less suitable for high-quality 3D reconstruction.

**Figure 3:**
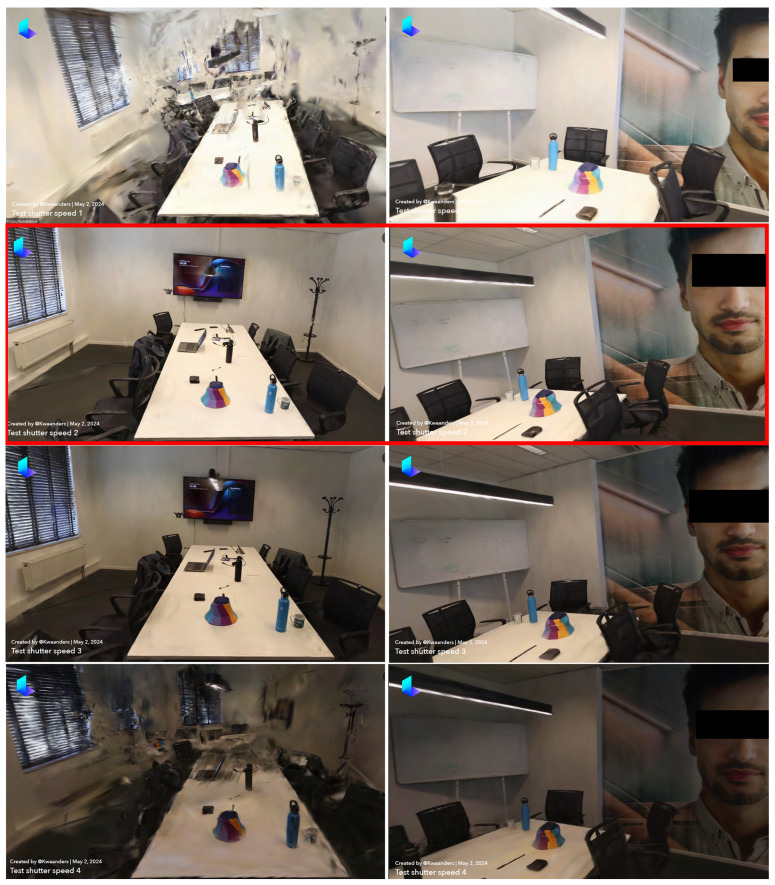
Shutter speed comparison—(**1**) 1/30, (**2**) 1/60, (**3**) 1/125, (**4**) 1/250.

In summary, the shutter speed of 1/60 s (Table 6) offers the best balance between noise, detail, and brightness for 3D reconstructions in indoor environments. This setting provides the clarity and accuracy necessary for detailed 3D models, making it ideal for crime scene investigations and other applications requiring precise 3D imaging, as illustrated in red in Figure 3.

By optimizing the shutter speed to 1/60 s, we could achieve the highest quality 3D reconstructions, enhancing the accuracy and utility of these models for various professional applications.

### 3.3. Comparison 3: Impact of Aperture Settings on 3D Reconstruction Quality

In this section, we analyze the impact of different aperture settings on the quality of 3D reconstructions. The aperture refers to the opening in a camera lens through which light passes to enter the camera. A larger aperture (indicated by a smaller f-number) allows more light to enter, resulting in brighter images with a shallow depth of field. Conversely, a smaller aperture (indicated by a larger f-number) allows less light, resulting in darker images with a greater depth of field. The aperture settings tested were f/3.5, f/4.5, f/5.6, and f/8, with all other parameters kept constant (ISO 400, shutter speed 1/60, and 24 FPS). The reconstructions were evaluated based on three criteria: the noise in the image, the level of detail in the reconstruction, and the brightness of the reconstruction compared to the actual room brightness. The comparison of these settings, along with all criteria and results, is summarized in Table 7.

When examining the noise, significant differences were observed among the reconstructions. The reconstructions at f/3.5, f/4.5, and f/5.6 were virtually noise-free, all scoring 5. These settings resulted in clear images with minimal noise, allowing for a clear visualization of the room’s features. In contrast, the reconstruction at f/8 scored a 1 due to the presence of noise, which significantly obscured the room’s features. Thus, in terms of noise, f/3.5, f/4.5, and f/5.6 were superior.

The level of detail in the reconstructions also varied across different aperture settings. The reconstructions at f/3.5, f/4.5, and f/5.6 were highly detailed, each scoring 5. These images allowed for clear identification of objects and even small text, making them ideal for detailed 3D modeling. The reconstruction at f/8, however, scored a 2 due to the significant loss of detail caused by noise. As such, the reconstructions at f/3.5, f/4.5, and f/5.6 were superior in terms of detail.

Brightness was another critical factor in this comparison. The reconstruction at f/8 was excessively dark, scoring a 1. The reconstruction at f/5.6 was also on the darker side, scoring a 3. In contrast, the reconstructions at f/3.5 and f/4.5 closely matched the actual room brightness, scoring 5 and 4, respectively. While f/3.5 was slightly brighter and f/4.5 slightly darker, both were close to the actual environment’s brightness levels. Therefore, in terms of brightness, f/3.5 and f/4.5 were effective.

Considering all three criteria, reconstructions at f/3.5, f/4.5, and f/5.6 emerged as the highest quality. However, reconstructions at f/3.5 provided the best balance between brightness, detail, and minimal noise, making it the optimal setting overall. Conversely, the reconstruction at f/8 was significantly inferior due to excessive noise and poor brightness.

**Figure 4:**
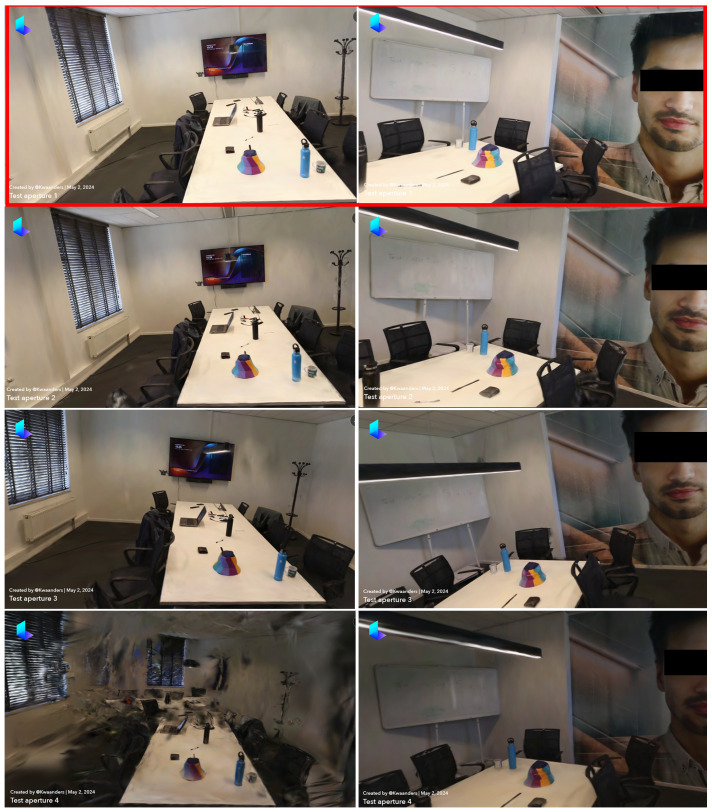
Aperture comparison—(**1**) f/3.5, (**2**) f/4.5, (**3**) f/5.6, (**4**) f/8.

In summary, an aperture setting of f/3.5 offers the best balance between noise, detail, and brightness for 3D reconstructions in indoor environments. This setting provides the clarity and accuracy necessary for detailed 3D models, making it ideal for crime scene investigations and other applications requiring precise 3D imaging, as illustrated in Figure 4.

By optimizing the aperture to f/3.5 (Table 7), we can achieve the highest-quality 3D reconstructions, enhancing the accuracy and utility of these models for various professional applications.

### 3.4. Summary and Recommendations

The comparative analysis of different camera settings (Table 8) revealed that ISO 200, a shutter speed of 1/60 s, and an aperture of f/3.5 provided the optimal balance for high-quality 3D reconstructions in our indoor environment. These settings minimized noise and artifacts, maximized detail, and ensured appropriate brightness levels, closely matching the actual room conditions.

These findings offer initial insights into achieving high-quality 3D reconstructions within controlled indoor environments, such as crime scene investigations. While our experiments were conducted in a single setting, we believe these results are applicable to similar indoor scenarios. However, further validation is needed across a variety of environments to ensure broader applicability. In particular, the camera settings we identified may serve as a baseline for professionals aiming to enhance the accuracy and effectiveness of their 3D models in controlled environments. A more comprehensive discussion, including comparisons with the existing literature, is presented in the following sections to support the generalizability of these findings.

## 4. Discussion

This section discusses the implications of the results obtained from the comparative analysis of various camera settings in respect to 3D reconstruction quality. The focus is on understanding how different settings influence noise levels, detail accuracy, and brightness, and how these findings compare with the existing literature. Additionally, the section provides recommendations for optimal camera settings based on the results and explores the potential applications and limitations of the study.

### 4.1. Comparison with the Existing Literature

The findings of this study are consistent with the existing literature on the impact of camera settings on image quality and 3D reconstruction. For instance, while some studies show that an ISO ranging from 200 to 400 is appropriate regarding sensitivity and noise, Quan and Li [34] discovered that a lower ISO means less noise, which leads to underexposure, and high ISO generates more noise, thereby losing the accuracy of details. The results also confirmed this, as our findings indicated that the best balance between noise reduction and exposure was met at ISO 200.

From the work of Mikamo, Furukawa and Kawasaki, a medium shutter speed range of 1/60–1/125 s was identified to be very important in reducing the amount of motion blur while still allowing the camera to obtain enough light. We also found that at a speed of 1/60 s on the shutter, the balance of reduced noise and limited motion blur was achieved without having the scene underexposed. On the other hand, higher shutter speeds reduced motion blur but underexposed the sense of detail visibility.

Our research extends the existing body of knowledge by applying these principles specifically to the context of 3D reconstruction for indoor crime scene investigations. While our experimental setup was a controlled environment, it was carefully chosen to simulate key features typically encountered in crime scenes, such as varied textures, objects of different sizes, and a mixture of natural and artificial lighting. For example, the inclusion of items like tables, wall-mounted objects, and complex surfaces, such as whiteboards, mirrors common elements found in crime scenes where evidence is distributed across different surfaces and planes. The consistency of our findings with the existing literature strengthens the argument that these results are applicable to crime scene reconstructions in similar indoor environments. However, further research across different crime scenes would help in verifying the generalizability of our conclusions.

### 4.2. Potential Applications

The results obtained from this research are of great relevance to many fields that require high-quality 3D reconstructions. In crime scene investigations, accurate 3D models can provide crucial insights into the sequence of events and spatial relationships between different pieces of evidence. The recommended camera settings can help investigators capture detailed and accurate reconstructions, enhancing the analysis and presentation of evidence.

Apart from forensic applications, the research findings may also enhance architecture, archaeology, and cultural heritage preservation. Accuracy in 3D construction is critical for the archaeological field and the preservation of cultural heritage. These reconstructions aid in the non-invasive documentation and analysis of historical sites and artifacts, supporting restoration efforts and enhancing public engagement through immersive virtual experiences. The high-quality reconstructions made possible by optimized camera settings are crucial for accurately documenting and preserving cultural heritage.

In virtual reality (VR) and augmented reality (AR) applications, high-quality 3D reconstructions enhance the realism and immersion of experiences. Improved training simulations, gaming environments, and educational tools benefit from detailed 3D models. By using the recommended camera settings, developers can ensure that the 3D models integrated into VR and AR applications are of the highest quality, providing users with a more engaging and immersive experience. This study broadens the scope of 3D reconstruction technology’s applicability across various professional fields, ensuring clear, detailed, and accurate models.

### 4.3. Limitations and Future Research

#### 4.3.1. Limitations

While this study provides valuable insights into the impact of camera settings on 3D reconstruction quality, there are some limitations to consider. The study was conducted in a controlled indoor environment, and the results may not be directly applicable to outdoor or more complex environments. Moreover, the type of objects and materials present in the scene were not varied significantly, potentially limiting the generalizability of the results to other types of scenes or objects with different reflective properties and textures. Future research should explore the impact of camera settings in different lighting conditions and environments to validate and extend these findings.

Additionally, the study focused on a specific set of camera parameters: ISO, shutter speed, and aperture. Even though these represent necessary factors that can determine the quality of an image, other factors like the quality of the lens and the size of the sensor, along with post-processing techniques, greatly influence the outcome in 3D reconstruction. The exclusive focus on these three parameters means that the findings provide a partial view of the factors influencing 3D reconstruction quality.

#### 4.3.2. Future Research

Future work could take the present results one step further by considering the influence of camera settings under an expanded range of conditions, such as outdoor scenarios, mixed lighting, and dynamic scenes. This would also validate the present findings and make them applicable in different real-world scenarios.

Moreover, future studies should consider the influence of other camera parameters such as lens quality, sensor size, and resolution. High-quality lenses and larger sensors can enhance image clarity and reduce noise, potentially leading to better 3D reconstructions. Exploring different types of cameras, such as those with full frame versus cropped sensors, can provide deeper insights into optimizing camera setups for specific applications.

In the future, the effects of other camera parameters, such as lens quality, sensor size, and resolution, should be considered. High-quality lenses and large sensors might improve image clarity and reduce noise to some extent, which could possibly result in better 3D reconstruction. Exploring different types of cameras, such as those with full frame versus cropped sensors, can provide deeper insights into optimizing camera setups for specific applications.

The role of post-processing techniques in improving 3D reconstruction quality should also be investigated. Techniques such as image stabilization, noise reduction, and contrast enhancement can significantly affect the final output. Understanding how these techniques interact with initial camera settings can help develop comprehensive guidelines for 3D reconstruction.

Furthermore, research may be directed toward developing automatic systems where the camera settings are adjusted at runtime based on conditions. Machine learning algorithms could make dynamic settings per frame for optimal settings, thus improving the quality and consistency of 3D reconstructions in varying conditions.

By addressing these areas, future research can further enhance the understanding of how to optimize 3D reconstructions for different applications that can improve not only the theoretical background but also practical realizations in fields like crime scene investigation, architecture, or cultural heritage preservation. This will guarantee the constant development and wider applicability of 3D reconstruction technologies.

## 5. Conclusions

This research systematically analyzed the influences of various camera settings—ISO, shutter speed, and aperture—on the quality of a 3D reconstruction in an indoor environment representative of a crime scene. By conducting controlled experiments, we identified optimal settings that minimize noise and artifacts, maximize detail accuracy, and ensure appropriate brightness levels in the reconstructed 3D models. Our results not only confirm findings in the literature for other indoor environments but also provide practical guidelines tailored to forensic applications.

These findings underscore the importance of camera settings in improving the accuracy and reliability of 3D reconstructions in forensic investigations. By offering a clear methodology, this research bridges the gap between theoretical insights and practical applications. In crime scene investigations, the ability to generate highly detailed 3D models enables investigators to preserve evidence with greater precision, reducing potential loss of information during on-site documentation. Beyond forensics, the insights provided here can guide professionals in other domains to optimize their workflows, thereby ensuring consistent and high-quality outputs.

The results suggest that an ISO of 200 is the best setting for balancing noise reduction with an acceptable level of exposure. Keeping the shutter speed at 1/60 s avoids any motion blurring within the image while still allowing enough light to enter. An f/3.5 aperture setting passes the most light into the camera and produces bright and detailed images with minimum noise. These settings allow the camera to capture every aspect in detail, and the detail of those minor components turns out to be crucial for creating a detailed 3D model of the reconstructed scene. This detail is important for an effective and accurate examination of the scene during crime investigation.

While the methodology developed in this study is specifically tailored for forensic applications, it also has potential for fields such as architecture, archaeology, and digital media, where high-quality 3D reconstructions are essential. For instance, these settings can improve artifact documentation in archaeology or facilitate precise interior modeling in architecture. The results demonstrate that optimizing camera settings directly influences the usability and fidelity of 3D reconstructions in diverse professional contexts.

Our findings are limited to controlled indoor settings and to a highly specific range of camera parameters. Future research needs to look at the influence of such settings in different scenarios, outdoors and indoors, under various lighting conditions, and needs to investigate more factors, such as lens quality, sensor size, and post-processing techniques. Moreover, an automated approach setting the proper camera settings in real time could produce 3D constructions of even better quality and make the 3D reconstruction process more efficient. Such advancements would broaden the applicability of this work, bridging the gap between laboratory conditions and real-world forensic and professional scenarios.

## Figures and Tables

**Table 1:** Comparison 1: Camera parameters.

Test #	ISO (A)	Shutter Speed (B)	Aperture (C)	FPS (D)
1.1	100	1/50	f/4	24
1.2	200	1/50	f/4	24
1.3	400	1/50	f/4	24
1.4	800	1/50	f/4	24

**Table 2:** Comparison 2: Camera parameters.

Test #	ISO (A)	Shutter Speed (B)	Aperture (C)	FPS (D)
2.1	400	1/30	f/4	24
2.2	400	1/60	f/4	24
2.3	400	1/125	f/4	24
2.4	400	1/250	f/4	24

**Table 3:** Comparison 3: Camera parameters.

Test #	ISO (A)	Shutter Speed (B)	Aperture (C)	FPS (D)
3.1	400	1/60	f/3.5	24
3.2	400	1/60	f/4.5	24
3.3	400	1/60	f/5.6	24
3.4	400	1/60	f/8	24

**Table 4:** Criteria for comparative analysis.

Criteria	1	2	3	4	5
Noise	There is too much noise present, and nothing can be seen	There is too much noise present, but the room is visible	There is some noise present, however, the outline of the room is still visible	Almost no noise is present, and the room is quite clear in visibility	There is no noise
Details	The reconstruction appears pixelated, yet it is discernible that an object should be present in that location	The reconstruction is pixelated, but it is still possible to discern the object type (e.g., table, chair, paper)	Identification of the object types is easily achievable	Capable of accurately identifying the object and providing brand information	Extremely detailed; there is no discernible difference between the model and the video
Brightness	The brightness is either too dark or bright, making it difficult to perceive any contrast	The brightness is either too dark or too bright, making it difficult to perceive	Brightness is okay but contrast is still lacking	Brightness is good, almost equal to the moment of capture	Brightness is excellent

**Table 5:** Comparison of ISO settings.

Criteria	Reconstruction 1.1 (ISO 100)	Reconstruction 1.2 (ISO 200)	Reconstruction 1.3 (ISO 400)	Reconstruction 1.4 (ISO 800)
Noise	1	2	2	2
Details	2	3	2	2
Brightness	1	4	5	2

**Table 6:** Comparison of shutter speed settings.

Criteria	Reconstruction 2.1 (1/30 s)	Reconstruction 2.2 (1/60 s)	Reconstruction 2.3 (1/125 s)	Reconstruction 2.4 (1/250 s)
Noise	2	4	5	1
Details	3	5	5	2
Brightness	4	4	3	1

**Table 7:** Comparison of aperture settings.

Criteria	Reconstruction 3.1 (f/3.5)	Reconstruction 3.2 (f/4.5)	Reconstruction 3.3 (f/5.6)	Reconstruction 3.4 (f/8)
Noise	5	5	5	1
Details	5	5	5	2
Brightness	5	4	3	1

**Table 8:** Optimal camera settings.

Parameter	Optimal Setting
ISO	200
Shutter Speed	1/60 s
Aperture	f/3.5

## Data Availability

Data is contained within the article.
